# Weak Genetic Isolation and Putative Phenotypic Selection in the Wild Carnation *Dianthus virgineus* (Caryophyllaceae)

**DOI:** 10.3390/biology12101355

**Published:** 2023-10-23

**Authors:** Jacopo Franzoni, Giovanni Astuti, Lorenzo Peruzzi

**Affiliations:** 1 PLANTSEED Lab, Department of Biology, University of Pisa, 56127 Pisa, Italy; lorenzo.peruzzi@unipi.it; 2 Botanic Garden and Museum, University of Pisa, 56126 Pisa, Italy; giovanni.astuti@unipi.it

**Keywords:** elevation, evolution, Fst, Mediterranean, morphometry, IBD, IBE, Pst, selection

## Abstract

**Simple Summary:**

Natural selection, genetic drift, and migration mainly drive evolution within species. By studying intraspecific variation at different elevations within the wild carnation *Dianthus virgineus*, we found a low genetic isolation paralleled by a high phenotypic differentiation among populations. In this species, genetic variation is related to geographic distance, whereas phenotypic variation does not result from genetic drift or gene flow limitations. Accordingly, we hypothesize that the phenotypic variation in this species is driven by selective forces, which act despite the presence of gene flow.

**Abstract:**

By relating genetic divergence at neutral loci, phenotypic variation, and geographic and environmental distances, it is possible to dissect micro-evolutionary scenarios involving natural selection and neutral evolution. In this work, we tested the patterns of intraspecific genetic and phenotypic variation along an elevational gradient, using *Dianthus virgineus* as study system. We genotyped genome-wide SNPs through ddRAD sequencing and quantified phenotypic variation through multivariate morphological variation. We assessed patterns of variation by testing the statistical association between genetic, phenotypic, geographic, and elevational distances and explored the role of genetic drift and selection by comparing the Fst and Pst of morphometric traits. We revealed a weak genetic structure related to geographic distance among populations, but we excluded the predominant role of genetic drift acting on phenotypic traits. A high degree of phenotypic differentiation with respect to genetic divergence at neutral loci allowed us to hypothesize the effect of selection, putatively fuelled by changing conditions at different sites, on morphological traits. Thus, natural selection acting despite low genetic divergence at neutral loci can be hypothesized as a putative driver explaining the observed patterns of variation.

## 1. Introduction

The micro-evolutionary forces acting within a species are mainly neutral processes, migration, and natural selection [1]. As a general mechanism, a reduction in gene flow among conspecific populations would promote genetic drift and impose less constraint on natural selection [2]. Nonetheless, selective processes are not incompatible with a low degree of isolation, as it is known that divergent selection may occur even though populations are linked by gene flow and divergence at neutral loci is low [3].

Relating neutral genetic variation to geographic and environmental differences sheds light on the mechanisms involved in the differentiation of natural populations [4]. In particular, when gene flow is spatially limited, the probability of gene exchange decreases with increasing geographic distance among populations, producing an isolation-by-distance pattern (IBD; [5,6]). A positive correlation between neutral genetic differentiation and geographic distances among populations, irrespective of environmental differences, may evidence the presence of an IBD, as shown for many animal and plant species [7,8]. On the other hand, when environmental differences across a species range positively covary with genetic differentiation among populations, independently from geographic distance, an isolation-by-environment pattern may emerge (IBE; [9]). Such patterns, typically characterizing species distributed across heterogeneous habitats [10,11], can be maintained by different mechanisms, including local adaptation to specific ecological conditions and non-random mating driven by environmentally induced phenotypic and phenological differences [6].

The detection of phenotypic patterns of variation, and their association with genetic variation at neutral loci, can also help to understand micro-evolution within species [12]. When phenotypic and genetic variations at neutral loci are positively associated, and both follow geographic distances, restricted gene flow may be the main factor shaping intraspecific variation [6,13]. A correlation between neutral genetic variation and phenotypic variation could also suggest that strong selection occurred, signifying the role of adaptive phenotypic divergence in restricting gene flow and maintaining genetic isolation [14,15,16]. This scenario would be further supported if phenotypic differences covary with the environment among populations. Alternatively, the absence of correlation between genetic variation at neutral loci and phenotypic differentiation among populations, paralleled by the detection of an IBE and high genetic differentiation, may point to incipient ecological differentiation due to local selection [15,17]. On the other hand, if populations show weak genetic structure and there is selection under gene flow, phenotypic variation should be independent from genetic divergence at neutral loci [3,12,18,19].

In this context, plant species distributed along broad elevation ranges are natural study systems that provide insights into the patterns and mechanisms of intraspecific variation [1,20]. Typically, plant populations growing at high elevations express a reduced size, thicker leaves, and higher tolerance to frost-induced stress than those growing at lower altitudes [21,22]. Indeed, elevational gradients are associated with drastic environmental changes (e.g., decreasing temperatures and increasing precipitations) in a relatively restricted geographic area [21], providing strong selective pressure that can promote divergence linked to local adaptation [23] or plastic phenotypic responses [18]. Moreover, elevational morphotypes and ecotypes can be maintained despite strong gene flow [18,19].

A feasible way to infer the presence of local adaptation in natural populations is to compare indices of neutral genetic differentiation (Fst) and indices of between-population differentiation of quantitative traits measured in wild populations (Pst) [24,25,26]. The rationale behind this approach is similar to that involving Qst, i.e., observed differentiation of quantitative traits between populations in a common garden; [27,28,29], but applied to trait measures obtained in the field. Fst is taken as a proxy for differentiation at neutral loci and provides a null hypothesis, reflecting genetic differentiation without selection [30]. Pst estimates the divergence of phenotypic traits among populations [26]. Pst-Fst comparisons can be interpreted similarly to Qst-Fst, albeit taking into account two possible pitfalls. First, variance components cannot be imputed to every environmental condition causing it [31]. Second, while calculating Pst, the components of additive genetic variance within (h^2^) and among populations (c) cannot be measured but only assumed [26]. However, Pst-Fst approaches can still be considered useful to explore the relation between environmental and phenotypic variation among populations, computing Pst for a range of c and h^2^ values and considering Pst-Fst comparisons as a tool to generate hypotheses on selection but not to test them. Thus, if Pst ≈ Fst, genetic drift can be imputed as the main differentiation mechanism for the trait for which Pst is calculated; if Pst is significantly different from Fst, either a divergent (if Pst > Fst) or stabilizing (if Pst < Fst) selection can be hypothesized [26]. Although there are limits to the usage of Pst over Qst [26,31], the former is a reliable way to determine whether genetic drift can be the sole driver of phenotypic differentiation, and it has been used in many recent works dealing with plant and animal species (e.g., [32,33,34,35]).

In this work, we explored the patterns of intraspecific genetic and phenotypic variation along an elevational gradient using *Dianthus virgineus* L. [36], an outcrossing perennial plant species, as the study system. *Dianthus virgineus* is a Mediterranean species of wild carnations, mainly distributed in southern France and the Italian Peninsula, occurring along a wide elevational range [37,38,39]. Populations of *D. virgineus* belong to a genetically uniform, recently originated (114–132 kya) evolutionary lineage [40]. In central Italy, botanists have historically observed qualitative morphological differences between lowland and montane plants [41]. Nevertheless, patterns of genetic and phenotypic variation have never been explored quantitatively, and the putative role of selection and gene flow has never been assessed in this widespread species.

To infer genetic relationships and structure, we genotyped genome-wide, neutral SNPs through ddRAD sequencing. To quantify phenotypic variation, we characterized multivariate morphological variation. Then, we assessed patterns of variation by testing statistical association between genetic, phenotypic, geographic, and elevation distances and explored the role of selection and genetic drift in phenotypic variation by comparing the Fst and Pst of the morphometric traits. In particular, if spatially restricted gene flow among populations is the main factor shaping intraspecific variation, we expect an association between phenotypic, neutral genetic, and geographic distances (IBD), but not with elevation, assuming that elevation and geographic distances do not covary. In this case, we also expect that the Pst values are not significantly different from the Fst of neutral loci. Alternatively, if environmental differences related to increasing altitude actually impose selection, we expect to retrieve a positive correlation between phenotypic variation and elevation. Moreover, this scenario would be supported by significant differences between Pst and Fst and a covariation between genetic fixation and phenotypic variation if ecological differentiation has happened. On the other hand, a statistical independence between these factors, combined with Pst values higher than Fst, would suggest that genetic drift is not the main force shaping phenotypic variation and that selection could be strong enough to overcome gene flow.

## 2. Materials and Methods

### 2.1. Study System

*Dianthus virgineus* belongs to a species complex of 21 morphologically similar taxa occurring in open habitats of the central Mediterranean area along a wide elevational range [42]. All species belonging to this complex are marked by branched woody stocks, flowers arranged in loose cymes or unbranched stems, and epicalyx scales abruptly contracted into a short mucro, 3–4 times shorter than the calyx [38,43]. In this group, reproduction mainly occurs through Lepidopteran-mediated outcrossing, favoured by proterandry, gynomonoecy, and gynodioecy [44,45,46].

### 2.2. Study Area and Sampling

**Figure 1:**
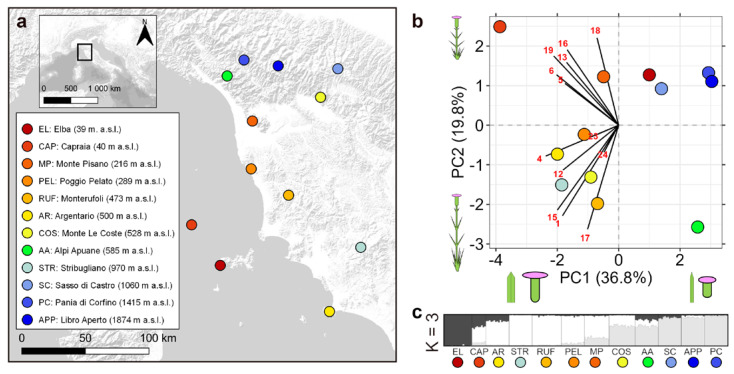
Patterns of overall geographic, phenotypic, and genetic variation among studied populations of *Dianthus virgineus*. (**a**) Study area and geographic distribution of the sampled populations, including elevations; (**b**) scatter plot of the first two components resulting from the Principal Component Analysis (PCA) on the refined morphometric dataset (for variable loading codes, see Table 1; the variation in basal leaves length and calyx length along PC1 and the variation in plant height along PC2 are graphically represented); (**c**) major-mode barplots at K = 3 (highest ΔK) resulting from customized STRUCTURE analysis on the genetic matrix (populations are arranged according to increasing latitude, with the exception of EL and CAP).

We selected as the study area a Mediterranean region from central Italy (Figure 1a), in which the geographical distribution of the target species is well known [47]. This relatively small geographic extension is characterized by elevational differences up to 2000 m. Along this elevational gradient, the annual mean temperature decreases with increasing elevation as well as the seasonality of the precipitation [48]. We collected data from 12 populations from June to September 2020, spanning from the coasts of Tuscan Archipelago (39 m a.s.l.) up to the Apennine mountains (1874 m a.s.l.) (Table S1). In the selected sampling sites, *D. virgineus* grows in populations composed of a high number of adult individuals (usually > 200), occupying a broad area of occupancy; nevertheless, we found a lower number of narrowly distributed flowering individuals (<50) on Capraia Island. With the exception of the latter locality, we collected material from 20 randomly selected adult individuals per sampling site. All these populations were assessed as a diploid with 2*n* = 2*x* = 30 chromosomes [49]. Populations were sampled during anthesis to collect parts of flowering individuals for morphometric and genetic analyses. After the collection, herbarium vouchers were prepared for all individuals sampled for morphometrics and leaves were silica-dried for DNA extraction.

### 2.3. DNA Extraction, Libraries Preparation, and Sequencing

The genetic analyses relied on genome-wide single nucleotide polymorphisms (SNPs) obtained using double digest restriction associated DNA sequencing (ddRAD-seq) [50]. Genomic DNA was extracted from silica-dried leaves using the adapted “sbeadex maxi plant kit” (LGC genomic, Hoddesdon, UK) and a KingFisher Flex Purification System (Thermo Scientific, Waltham, MA, USA). We extracted the DNA of 172 individuals, and 2 individuals per plate were processed in triplicate (positive controls). Then, 10–20 mg of silica-dried leaf samples were randomly distributed in two 96-well plates, finely ground for 4 min using a Retsch-Mill (Retsch, Haan/Duesseldorf, Germany) and metal beads, and incubated with Lysis Buffer PN (350 µL per sample), thioglycerol (3.5 µL per sample), and DCB (10 µL per sample) at 65 °C and 1000 rpm for at least 60 min in an Eppendorf ThermoMixer. After centrifugation, 200 µL of the resulting supernatant for each sample was added to a binding solution (400 µL of Binding Buffer PN, 9 µL of sbeadex particle suspension). The extraction process was performed by loading the KingFisher machine with one binding plate (containing lysed samples), two Washing Buffer PN1 plates (400 µL per sample), one Washing Buffer PN2 plate (400 µL per sample), and one elution plate (100 µL of AMP buffer per sample). The concentration of extracted DNA was quantified on a Spark plate reader (Tecan, Männedorf, Switzerland) using the Quantifluor ONE dsDNA kit (Promega, Madison, WI, USA).

The library preparation followed the protocol used by Westergaard et al. [51]. Briefly, the digestion of 100 ng of high-quality genomic DNA was carried out in a 25 μL reaction volume with 0.4 μL EcoRI-HF (20 U) and 2.5 μL Buffer CutSmart (New England Biolabs, Inc.) for 30 min at 37 °C, followed by 0.4 μL Taqα1 (New England Biolabs, Inc., Ipswich, MA, USA) for 30 min at 65 °C. The double digest was ligated to adaptors in a 30 μL reaction volume using 2 μL P1 Adapter, 2 μL P2 Adapter, 0.8 μL T4 DNA ligase buffer (10×) and 1 μL T4 DNA ligase (400 U/μL). Forty-eight individually barcoded samples were multiplexed in a pooled library. Further, 500–550 bp libraries were selected using the first 0.57× AMPure beads and subsequently 0.12× AMPure beads. This size selection step also removes unligated adapters. The libraries were then washed while attached to 15 µL Dynabeads M-270 Streptavidin beads (Invitrogen, Waltham, MA, USA) to select for P2-biotin-labelled adapters. Illumina flowcell annealing sequences, unique double-index primers, multiplexing indices, and sequencing primer annealing regions were added to each library during the PCR amplification performed with a KAPA Hifi Hotstart ready mix (Kapa Biosystems, Inc., Wilmington, MA, USA) for 8–10 cycles. The libraries were further cleaned using AMPure XP beads and checked for DNA quantity on a Quantus using the kit and for optimal fragment sizes on a Tapestation 2200 (Agilent, Santa Clara, CA, USA) using the HS D1000 tape. Sets of two libraries were multiplexed and sequenced in two lanes of 150-bp paired-end reads on an Illumina NovaSeq 6000 at Novogene UK (Cambridge, UK).

### 2.4. Reference Assembly, Mapping, SNPs Calling, and Filtering

Raw sequences were demultiplexed using the process_radtags component of STACK v.1.26 [52] with -q option to filter out reads with a Phred Quality Score lower than 10. De novo reference assembly, read mapping, and variant calling were performed following the dDocent pipeline [53]. A reference was built using reads from the studied populations and from individuals collected from the Balkans, Alps, and Southern Italy [40] in order to work with a genetically representative reference of the *D. virgineus* complex. The dDocent pipeline concatenates forward and reverse reads to generate sets of unique sequences that are then clustered into reference contigs by the software RAINBOW 2.0.4 [54] and CD-HIT 4.8.1 [55]. The reference assembly used unique reads with a coverage higher than 4 (discarding likely sequencing errors) and occurring in more than 4 individuals (eliminating non-informative sequences at a population level). As highlighted by Westergaard et al. [51], two important parameters that can affect reference assembly are the number of reads set to retain unique sequences (K) and the threshold similarity used by CD-HIT to cluster sequences (-c). The final assembled reference was obtained by setting K = 10 and −c = 0.9 and was composed of 33,518 contigs. The reads from samples were mapped on the reference with BWA-MEM [56] with default settings, eliminating mapped reads with a mapping quality score below 10 and PCR duplicates with SAMTOOLS [57]. In the resulting BAM files, variants were called with FREEBAYES v 1.1 [58], producing a raw VCF (Variant Call Format) file containing 1,005,306 called variants. The raw VCF file was filtered following the dDocent pipeline (https://github.com/jpuritz/dDocent/blob/master/tutorials/Filtering%20Tutorial.md (accessed on 15 November 2020). The variants called in 50% of the individuals and with a minimum quality score of 30, a minor allele count of 3, and a minimum depth for a genotype call of 3 were initially kept. Four different individuals belonging to 4 different populations were discarded from the dataset due to a percentage of missing data higher than 45%. Variants with a 0.7 call rate and a minor allele frequency of 0.05 were kept. Additional filters to remove variants resulting from sequencing errors, paralogues, multicopy loci, or artefacts of library preparation were applied, as recommended in the pipeline. After keeping only single nucleotide polymorphism (SNP) variants, loci with significantly different allelic frequencies from the null model of Hardy–Weinberg equilibrium (*p* < 0.001, according to Wigginton et al. [59]) within each population were discarded. Finally, to avoid a linkage disequilibrium of the SNPs, the dataset was pruned with BCFTOOLS [60], eliminating loci with r^2^ values higher than 0.2 in a 1000-site frame. The mean genotyping error rate of the dataset was calculated with TIGER [61].

### 2.5. Genetic Structure

To explore the genetic structure of the studied populations, we inferred the number of genetically homogeneous clusters (K) by performing a Bayesian cluster analysis with STRUCTURE 2.3.4 [62,63,64,65]. To avoid problems of low accuracy in the inference of K in STRUCTURE due to unbalanced sample dimensions among sampling sites, we performed our analyses following Wang [66]. Firstly, an explorative analysis was performed with default parameters, i.e., an ancestry model with admixture, a relative admixture level (α) equal to 1, no a priori information on sampling localities, and assuming the allelic frequencies among populations correlated. Then, we conducted an analysis with an alternative ancestry model, with an initial α value of 0.11 (α = 1/K, using the number of more likely groups obtained in the explorative analysis, i.e., 9) with a priori information on the sampling localities (LOCPRIOR), and using the correlated frequency model. We ran each analysis with 10 replicates for each value of K from 1 to 12, with a burn-in of 10,000 generations followed by 30,000 generations of the Markov–Monte Carlo chain (MCMC). The most likely number of genetic clusters (K) was inferred in STRUCTURE HARVESTER [67], using either the ΔK statistics according to Evanno et al. [68] or the calculation of the probability of obtaining the genotype X given K (Pr[X|K]; [62]). We aligned and visualized bar plots using the CLUMPAK (Cluster Markov Packager Across K) web server [69].

The genetic fixation among populations was assessed by building a pairwise F_ST_ matrix (calculated according to Nei [70]) with *hierfstat* package [71], and 99% confidence intervals were calculated using the boot.vc() function, bootstrapping 1000 times.

### 2.6. Morphometry

Morphometric analyses were carried out to reveal quantitative patterns of variation and to detect phenotypic differentiation in adult plants among populations. We measured 24 quantitative variables regarding vegetative organs (leaves and stems) and reproductive structures (corolla and petals, calyx, anthers, ovaries, epicalyx scales) (Table 1). These characters are known to vary among and within different species belonging to the *D. virgineus* complex [39,42,72]. Moreover, in many species of *Dianthus*, the variation in traits such as plant height, leaf dimensions, and calyx length is known to be associated with climatic variation and oscillations [39,72,73] and subjected to local adaptation, driven by abiotic factors, at different elevations [74]. Seven variables were measured on fresh materials, and the remaining on the same individuals after being prepared as herbarium specimens (Table 1). Raw data were acquired with an electronic caliper. Morphometric data were analysed using R ([75]; https://www.r-project.org/index.html (accessed on 1 September 2023) and PAST (https://www.nhm.uio.no/english/research/resources/past/ (accessed on 1 September 2023)).

**Table 1:** Measured morphometric variables in the studied populations of *Dianthus virgineus*. The description of the characters, their ID code used in the analyses, and the type of material on which they were measured (either on fresh material = F or on dried herbarium specimens = D) are reported.

Character (Unit of Measure)	ID	Fresh (F)/Dried (D) Material
Plant height (cm)	1	D
Number of internodes	2	D
Lower internode length (mm)	3	D
Upper internode length (mm)	4	D
Basal leaf length (mm)	5	D
Basal leaf width (mm)	6	D
Upper stem leaf length (mm)	7	D
Upper stem leaf width (mm)	8	D
Lower stem leaf length (mm)	9	D
Lower stem leaf width (mm)	10	D
Number of flowers per stem	11	D
Number of epicalyx scales	12	D
Upper epicalyx scale length (mm)	13	D
Upper epicalyx scale mucro length (mm)	14	D
Upper epicalyx scale width (mm)	15	D
Calyx length (mm)	16	D
Calyx width (mm)	17	F
Calyx teeth length (mm)	18	D
Corolla diameter (mm)	19	F
Petal length (mm)	20	F
Petal limb length (mm)	21	F
Petal limb width (mm)	22	F
Ovary length (mm)	23	F
Anther length (mm)	24	F

After data collection, we prepared the dataset for further multivariate analyses. Firstly, two individuals were removed from the dataset due to missing data (one from AR and one from CAP). Secondly, we removed variables that did not show significant differences among populations. We tested for the homoscedasticity of each variable with the Levene test (significance threshold α = 0.05), and then we performed univariate analyses on each morphometric character using an ANOVA or Kruskal–Wallis test and multiple comparison tests, Tukey–Kramer’s post hoc test, and Mann–Whitney’s U test with Bonferroni correction (α = 0.01), respectively. Thirdly, we built a dataset with mean values per population of each variable, and we filtered out highly correlated variables showing Pearson’s r >0.8. Finally, to avoid distortions in the multivariate analyses due to different absolute values, each variable was then standardized to z-score values. The overall variability in the refined dataset was explored with a Principal Component Analysis (PCA).

Spearman correlation tests between morphometric traits and elevations of populations were performed. In the case of a highly significant correlation (*p* < 0.01), to understand if elevation could be a good predictor of the variation in the trait, regression (linear and polynomial) analyses were carried out.

### 2.7. Mantel Tests

The Mantel test is a straightforward method to test biological hypotheses formulated as associations among distance matrices [76]. To test correlations among genetic structures (genetic fixation calculated as pairwise F_ST_), geographic and elevational distributions, morphometry, and the seed germination of the studied populations, Mantel tests (mantel.rtest() function from the *ade4* package; [77]) were performed, setting the number of iterations to 9999. The significance threshold was set at 0.01. The geographic distance matrix (in km) was calculated with the *geosphere* package [78]. The elevational distance matrix was calculated using the absolute values of differences among population elevations. To build the morphometric distance matrix, we calculated the Euclidean distance among populations using the dist() function on the z-score transformed and filtered dataset (see Section 2.6). In order to represent graphically the significant correlations between matrices, scatterplots were built and regression lines were interpolated.

### 2.8. Pst-Fst Comparisons

To estimate the differentiation of morphological traits among populations and to test the role of selection, we estimated Pst values using the *Pstat* R package [79]. For each trait, Pst was computed with the formula proposed by Brommer [26]:

(1) $$Pst=\frac{\frac{c}{h^{2}}\sigma_{B}^{2}}{\frac{c}{h^{2}}\sigma_{B}^{2}+2\sigma_{W}^{2}}$$

In (1) $\sigma_{B}^{2}$ and $\sigma_{W}^{2}$ are the between- and the within-population variance components of the target trait, respectively; h^2^ denotes the proportion of additive genetic effects on the variation within populations, whereas c is the proportion of additive genetic effects on the variation between populations. Contrary to Qst, when calculating Pst, no direct measurement of h^2^ and c can be obtained, and in a hypothetical scenario, c = h^2^ is assumed. Then, to understand if Pst can be used as a substitute for Qst, Pst is estimated using a range of c and h^2^ values [26]. Natural selection can be robustly argued if the Pst values are higher than Fst for a c/h^2^ close to zero, meaning that c is much smaller than h^2^, which is likely due to the presence of environmental heterogeneity among population sites [26]. Thus, the lower the c/h^2^ when Pst is significantly higher than Fst, the stronger the evidence of a putative role of selection in shaping the studied trait.

We calculated the Pst values for three morphological traits, selected according to the explorative multivariate analyses and the correlation test results with elevation. Firstly, we assumed that the proportion of additive genetic effects on the variation within and across populations did not differ (c/h^2^ = 1), also assuming that the former was higher than the latter (c/h^2^ = 0.1). We assessed the robustness of Pst-Fst comparison by calculating Pst for an interval of c/h^2^ values, spanning from zero to two. For each Pst estimation, 99% confidence intervals were calculated by bootstrap resampling 1000 times. Genetic drift is not considered to have a relevant contribution to phenotypic differentiation if the confidence intervals of the Fst and Pst estimations do not overlap for c/h^2^ ≤ 0.5 [26].

## 3. Results

### 3.1. Genetic Analyses

We retrieved a VCF file containing 654 biallelic SNPs across 184 samples (172 individuals, 4 of which were replicated three times), with 3.81% of the missing data and 2.22% of the mean genotyping error rate. The retained SNPs were unlinked and at Hardy–Weinberg equilibrium within each population.

According to Evanno’s statistics, an explorative STRUCTURE analysis (α = 1; no prior model on sampling localities; correlated allelic frequencies) suggests K = 9 as the number of the most homogeneous genetic clusters (ΔK = 147.28; Pr[X|K] = −116,491.09 ± 8987.42; Figure S1). In the customized analysis (α = 0.11; priors model on sampling localities; correlated allelic frequencies), K = 3 shows the highest ΔK value (ΔK = 96.01; Pr[X|K] = −114,236.56 ± 4.41), while K = 4 shows the highest Pr[X|K] value (ΔK = 4.91; Pr[X|K] = −113,593.69 ± 146.1; Figure S1). In the bar plot obtained with K = 3 (Figure 1c), we can detect the genetic distinctiveness of EL individuals (dark grey). Conversely, the southernmost and northernmost populations cluster in two different groups (indicated in Figure 1c by white and light grey barplots, respectively) that show admixture at intermediate latitudes. The geographic structuring of the dataset is also supported by the major mode (7/10) of the K = 4 structure (Figure S1), in which the two southernmost populations (AR and STR) belong to a separate cluster. The same pattern can be observed with the major mode (8/10) of K = 2 (ΔK = 45.26; Pr[X|K] = −115,302.42 ± 7.24; Figure S1).

The calculated pairwise Fst among populations (Table S2) varies from a minimum of 0.01657 (RUF vs. PEL) to a maximum of 0.07468 (EL vs. APP), with a mean value = 0.04864 ± 0.01255 standard deviation. The Fst values showed 99% confidence intervals spanning from 0.0435 to 0.0534, being significantly different from zero.

### 3.2. Morphometric Analyses

After the refinement of the morphometric dataset, 11 variables were removed (Table S3). The length of the epicalyx scale mucro and lower internode were filtered out, as they did not differ among populations (Table S4). The final morphometric dataset used for multivariate analyses contained 13 variables for 12 populations.

**Figure 2:**
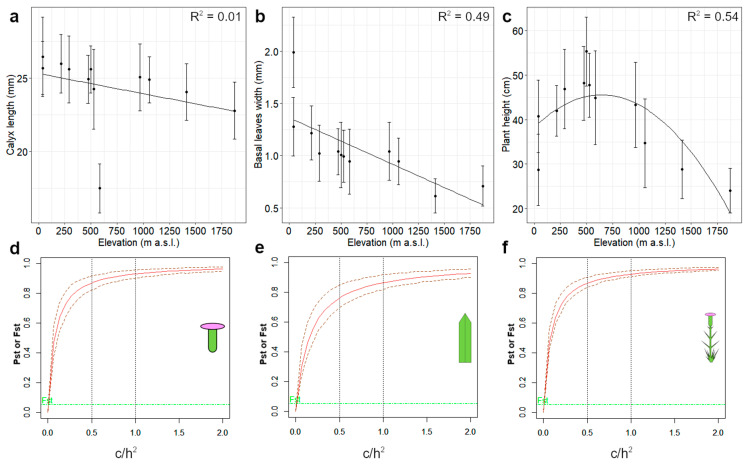
Variation with elevation and comparisons of phenotypic and neutral genetic differentiation of calyx length (**a**,**d**), plant height (**b**,**e**), and basal leaf width (**c**,**f**) in the studied populations of *Dianthus virgineus*; (**a**–**c**) relations of the three morphological features with elevation. Mean values (±SD) for each character is presented. Regression lines and adjusted R^2^ are shown; (**d**–**f**) Pst-Fst comparisons for a range of c/h^2^ values. Green lines represent the upper limit of 99% confidence intervals of Fst values. For Pst variation (red lines), 99% confidence intervals are shown as dashed lines.

In the PCA, the first two components explain 56.6% of the measured variation (Figure 1b). The morphometric traits were partitioned in two groups of correlated variables. The first group separates the populations of the upper left from the lower right quadrant (including 5, 6, 13, 16, 18, 19), and the second group separates the populations of the lower left from the upper right quadrant (including 1, 4, 12, 15, 17, 23, 24). According to variable loadings along PC1 (36.8%), all the studied traits are negatively correlated with this component, as the lowland populations, including taller plants with longer and wider leaves and flowers, longer calyxes, and wider corollas, show lower PC1 values than populations from Apennine (SC, PC, and APP), characterized by small-sized individuals with shorter and narrower leaves, short calyxes, and small corollas (Figure 2c). Surprisingly, the Elba island population (EL) is more similar to the Apennine populations than to plants from other coastal localities, whereas the Capraia island population (CAP) is relatively distant from the other studied populations. PC2 (19.8%) is positively related to traits characterizing CAP, i.e., basal leaf length and width, and negatively related to traits differentiating the Apuan Alps population (AA), i.e., calyx length and corolla diameter. In addition, plant height is negatively related with PC2, as populations including shorter plants are located at higher PC2 values.

The results of the Spearman’s correlation tests are listed in Table S5. The three morphological variables that show the highest significant correlation with elevation are basal leaf width (r_s_ = −0.86, *p* = 5.97 × 10^−4^; Figure 2a), calyx length (r_s_ = −0.84, *p* = 0.001; Figure 2b), and lower stem leaf width (r_s_ = −0.78, *p* = 0.005). However, basal leaf width and lower stem leaf width are highly correlated (r_s_ = −0.96, *p* = 0.02; Table S3). Elevation is a good predictor for basal leaf width according to the linear regression results (adjusted R^2^ = 0.49, F = 11.44, *p* = 0.007). Testing the association between calyx length and increasing elevation after removing the outlying AA populations results in a higher, negative, and significant correlation (r_s_ = −0.91, *p* = 5.55 × 10^−5^). Moreover, a linear model does not fit when considering the calyx length of AA (adjusted R^2^ = 0.01, F = 1.17, *p* = 0.30), whereas it shows a good fit when this population is excluded (adjusted R^2^ = 0.76, F = 33.12, *p* = 0.0003). Plant height is not linearly correlated with increasing elevation (rs = −0.22, *p* = 0.48), as EL shows values overlapping with those of high elevation populations (Figure 2c). However, according to a quadratic regression analysis, elevation explained a good amount of the plant height variation (adjusted R^2^ = 0.54, F = 7.46, *p* = 0.01).

### 3.3. Mantel Tests

The genetic, morphometric, elevational, and geographic distance matrices are shown in the “Supplementary materials” (Tables S2 and S6–S8).

**Figure 3:**
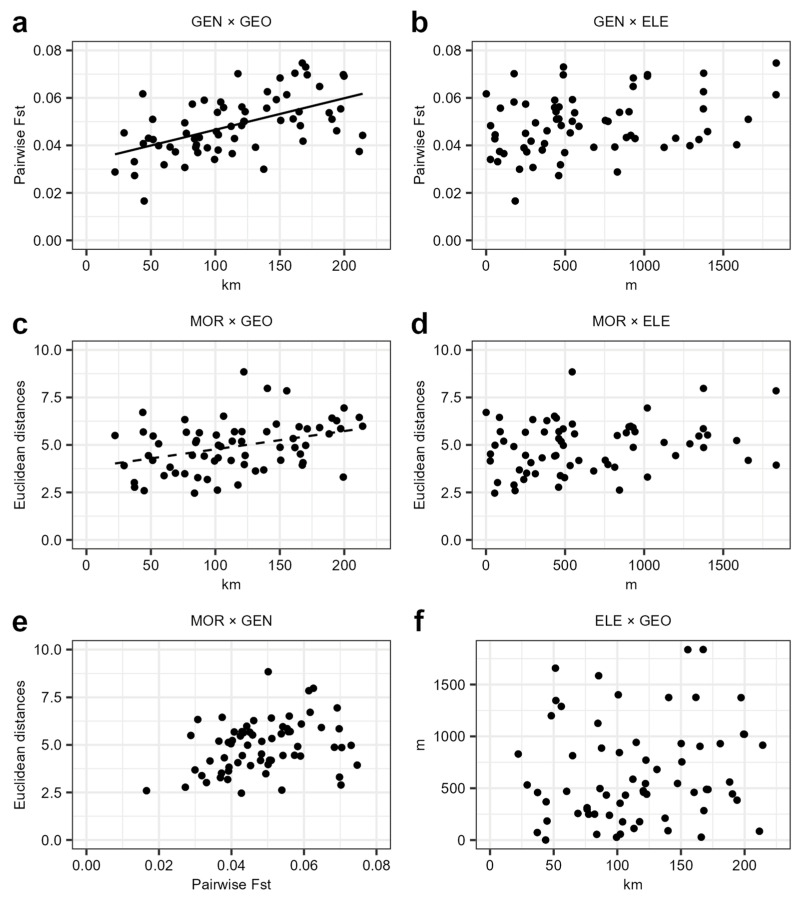
Relations between neutral genetic, phenotypic geographic, and elevational distance in *Dianthus virgineus*. Scatter plots of six Mantel tests are shown. Each point represents a pairwise comparison between two different populations. (**a**) Variation of pairwise genetic fixation of populations (GEN) with increasing geographic distance (GEO); (**b**) variation of GEN with increasing elevational distance (ELE); (**c**) variation of pairwise Euclidean distance of uncorrelated morphometric features (MOR) with ELE; (**d**) variation of MOR with ELE; (**e**) variation of MOR with GEN; (**f**) variation of ELE with GEO. For significant correlations with *p* < 0.01, entire regression lines are shown, whereas for significant correlations with *p* < 0.05, dashed regression lines are shown.

The genetic distance at neutral loci is significantly correlated with geographic distance among populations (r = 0.539, *p* < 0.01, Figure 3a), but not with elevational difference (r = 0.301, *p* = 0.138, Figure 3b). On the other hand, morphometric distance is not correlated with elevation distance (r = 0.238, *p* = 0.138, Figure 3d), but it show a positive correlation with geography, albeit slightly above the significance threshold (r = 0.362, *p* = 0.013, Figure 3c). No significant correlation was found when testing the association between genetic and morphometric distance among populations (r = 0.301, *p* = 0.100, Figure 3e). Elevational and geographical distance are not statistically significantly correlated (r = 0.125, *p* = 0.210, Figure 3f).

### 3.4. Pst-Fst Comparison

The morphological traits selected to perform the Pst-Fst comparison are calyx length, plant height, and basal leaf width. Calyx length and basal leaf width belong to the same group of correlated variables in the PCA and are significantly correlated with elevation. Plant height belongs to the other group of variables, and, although it is not linearly correlated with elevation, it is known to be under selection in plant populations growing at high altitudes [23].

Pst values and their 99% confidence intervals for the three considered morphological traits, assuming c/h^2^ = 1 and c/h^2^ = 0.1 (Table 2), are higher than those of Fst values. The highest Pst values at c/h^2^ = 1 are estimated for the differentiation of calyx length; however, plant height shows a narrower confidence interval (Table 2). Basal leaf width exhibits the lowest Pst values at c/h^2^ = 1 (Table 2). By computing multiple Pst values using a range of c/h^2^ values, we show that the phenotypic differentiation of these traits is higher than the genetic differentiation at neutral loci starting from very low c/h^2^ values, showing an overlap of Pst and Fst only for c/h^2^ values approaching zero (Figure 2d–f).

## 4. Discussion

After exploring and testing patterns of intraspecific variation along an elevation gradient in *D. virgineus*, we revealed a weak genetic structure related to geographic distance among populations, and we also excluded a relevant role of genetic drift acting on phenotypic traits. A high degree of phenotypic differentiation with respect to genetic divergence at neutral loci allowed us to hypothesize a role of selection, putatively fueled by changing conditions at different sites, on morphological traits. Thus, natural selection acting despite low genetic divergence at neutral loci can be argued as a putative scenario explaining the observed patterns of variation.

### 4.1. Patterns of Genetic Variation

The fixation indices of genetic differentiation at neutral loci among populations are very low, consistent with average Fst values obtained in other studies that underlie a weak genetic structure within plant species (Fst = 0.039 ± 0.008 SD; [6]). Thus, the STRUCTURE outputs should be interpreted as the result of a weakly structured genetic continuum across the study area, rather than of an actual separation between the groups of populations [80]. Indeed, considering the K = 3 scenario, we observed that the southernmost and northernmost continental populations cluster in two different groups, admixing populations from intermediate latitudes. Although the geographic distance among populations explains genetic variation, the very low Fst values do not support a true genetic isolation of the studied populations. Nevertheless, individuals from the Tuscan Archipelago, especially from Elba Island (EL), show a higher degree of genetic differentiation, likely due to insularity [81]. Moreover, considering the non-significant correlation between increasing elevation and genetic fixation, we could rule out a possible IBE pattern in shaping genetic variation at the neutral loci of the studied populations [4,14], in line with other studies along elevation gradients (e.g., [82]). Thus, all this evidence supports that the studied populations belong to a single genetic group (see also Luqman et al. [40]), only weakly structured according to geographical distance.

Our results suggest that the studied populations are connected through gene flow. Indeed, *D. virgineus* is widely distributed across the continental part of the study area [47]. The main pollinators of *Dianthus* are Lepidopterans [83], highly mobile insects that can move pollen over relatively large geographic areas [84]. In addition, the presence of gynomonoecy and gynodioecy, combined with proterandry [44,45,46], promote cross-pollination, increasing the probability of gene exchange between geographically close populations.

### 4.2. Patterns of Phenotypic Variation

The morphometric results point to the role of local environmental conditions in determining, possibly through selection, the phenotypic variation within *D. virgineus*. Although the overall morphometric variation is not statistically correlated with elevational distances among populations, we measured a negative association along the elevational gradient of some vegetative and reproductive traits, in line with a general trend of decreasing plant size with increasing elevation [21,22]. This pattern of morphometric variation supports the role of changing environmental conditions with increasing elevation in determining phenotypic differentiation in the genus *Dianthus*. For instance, high-elevation ecotypes, characterized by reduced plant size, have also been observed in *D. carthusianorum* L. in the Swiss Alps [74]. On broader geographic and evolutionary scales, a study correlating multivariate morphometric variation with environmental variability among genetically separated lineages in the *D. virgineus* complex proved that increasing elevation and decreasing temperature are negatively associated with the traits determining plant biomass (e.g., stalk size and leaf width) [39].

The Mantel test between overall morphometric and elevation distances was not significant, suggesting that other environmental factors, not directly related to the elevational gradient, are acting on the morphological variation in *D. virgineus*. For instance, the non-linear pattern of plant height (showing a very high Pst) with elevation suggests that there may be other biotic and abiotic factors contributing to the variation in plant size. Concerning reproductive traits, calyx length decreases with elevation and shows conspicuous Pst values, but it is significantly shorter in individuals from Apuan Alps (AA), collected at just around 600 m a.s.l. The size of calyx, and of flowers in general, is functionally connected with pollination ecology in *Dianthus* [85]. For instance, in the Alpine *D. inodorus* (L.) Gaertn., smaller flowers (often pistillate) are less likely to be affected by seed predators compared to larger, hermaphroditic flowers ([86], under the name *D. sylvestris* Wulfen). Accordingly, the reduced flower size observed in AA may be due to strong selection, as it could be advantageous to avoid a fitness decline due to seed predators. These hypotheses on putative selective pressures acting on the phenotypic variation in *D. virgineus* will require future in-depth testing.

### 4.3. Scenarios of Intraspecific Differentiation

Phenotypic differences among populations of *D. virgineus* are not correlated to geographic distance or genetic divergence at neutral loci. Although the overall morphometric variation is positively correlated with geographic distances, the differentiation of quantitative traits is much higher than the genetic differentiation at neutral loci (Pst > Fst). These results do not allow a major role of gene flow limitations and genetic drift in shaping phenotypic variation among populations to be hypothesized. On the other hand, our results could support an alternative hypothesis in which the phenotypic variation in *D. virgineus* is the result of divergent selective pressures that are strong enough to counteract gene flow [26,32,35]. A covariation of some morphological traits with elevation suggests, in some cases, that changing environmental conditions act as selective pressures. Indeed, the occurrence of selection despite low differentiation at neutral loci has been reported in other animal and plant species distributed along elevation gradients [12,18,19]. This conclusion is limited by the fact that we studied plants directly in their natural habitats. Indeed, phenotypic plasticity is a common phenomenon along elevation gradients in plants [87], and it has been documented in other species of the genus *Dianthus* [88]. Nonetheless, when comparing Pst and Fst at multiple c/h^2^ values, the Pst values for the three considered traits overlap with the Fst values at c/h^2^ values approaching zero, suggesting that phenotypic differentiation can be used as a robust proxy for Qst to infer putative natural selection [26]. In addition, plants regenerated from the seeds of our target populations are currently cultivated at the Botanical Garden of Pisa [89], and preliminary observations seem to rule out a significant role of phenotypic plasticity in shaping the morphological traits of this species.

## 5. Conclusions

For the first time, we provided evidence supporting the putative role of phenotypic selection acting despite low genetic divergence at neutral loci in the widespread Mediterranean species *D. virgineus*. Although the studied populations show a weak genetic structure, differentiation at neutral loci parallels geographic distance but not elevation. Moreover, the significantly higher Pst values of the selected morphometric traits with respect to the Fst values do not support the role of gene flow limitations and genetic drift in shaping the observed phenotypic variation. Alternatively, selection under gene flow can be hypothesized to be a possible scenario to explain the intraspecific variation in *D. virgineus*.

## Figures and Tables

**Table 2:** Pst-Fst comparison results for three morphometric traits of *Dianthus virgineus*. Pst were calculated assuming c/h^2^ = 1 and c/h^2^ = 0.1. For each Pst value and Fst value, 99% lower and upper confidence interval (CI) boundaries are reported.

Character	ID	c/h^2^	Pst/Fst	99% Lower CI	99% Upper CI
Plant height	1	1	0.929	0.903	0.957
		0.1	0.566	0.484	0.684
Basal leaf width	6	1	0.864	0.800	0.925
		0.1	0.388	0.285	0.555
Calyx length	16	1	0.930	0.887	0.961
		0.1	0.571	0.449	0.718
Genetic divergence at neutral loci			0.049	0.043	0.053

## Data Availability

Raw Illumina sequence data will be available in an online repository. The data (i.e., morphometric dataset, and filtered SNPs VCF file) that support the findings of this study are openly available in the Figshare digital repository at http://doi.org/10.6084/m9.figshare.20367279.

## Supplementary Materials

The following supporting information can be downloaded at https://www.mdpi.com/article/10.3390/biology12101355/s1: Figure S1: Major-mode barplots among the studied populations of *Dianthus virgineus*, resulting from STRUCTURE analyses; Table S1: Geographic, environmental, and sampling information of the studied populations of *Dianthus virgineus*; Table S2: Pairwise F_ST_ among the studied populations of *Dianthus virgineus*; Table S3: Correlation of morphometric traits among the studied populations of *Dianthus virgineus*; Table S4: Significantly different morphometric variables among the studied populations of *Dianthus virgineus*; Table S5: Spearman’s correlation test results between morphometric variables and elevation in the studied populations of *Dianthus virgineus*; Table S6: Morphometric distances among the studied populations of *Dianthus virgineus*; Table S7: Elevation distances among sampling sites of the studied populations of *Dianthus virgineus*; Table S8: Geographic distances among sampling sites of the studied populations of *Dianthus virgineus*.
