# Supplementary material for: Weak Genetic Isolation and Putative Phenotypic Selection in the Wild Carnation Dianthus virgineus (Caryophyllaceae)
Source: Biology (Basel). 2023 Oct 23;12(10):1355. doi: 10.3390/biology12101355 (PMC10604185; doi:10.3390/biology12101355)
Supplement: Supplementary file 1 [file biology-12-01355-s001.zip › biology-2674432-supplementary.pdf]

Figure S1. Major mode barplots among the population of *Dianthus virgineus* resulting from STRUCTURE analyses. Genetic matrix contained 654 unlinked SNP-genotypes and 184 samples, including 3 replicates of 4 individuals. Populations are arranged according to increasing elevation. Upper barplot: structure inferred for K = 9 (major mode 8/10; ΔK [highest] = 147.28; Pr[X|K] = -116491.09 ± 8987.42) output from the explorative analysis. Middle barplot: structure inferred for K = 2 (major mode 8/10; ΔK = 45.26; Pr[X|K] = -115302.42 ± 7.24) output from the customized analysis. Lower barplot: structure inferred for K = 4 (major mode 7/10; ΔK = 4.91; Pr[X|K] [highest] = -113593.69 ± 146.1) output from the customized analysis.

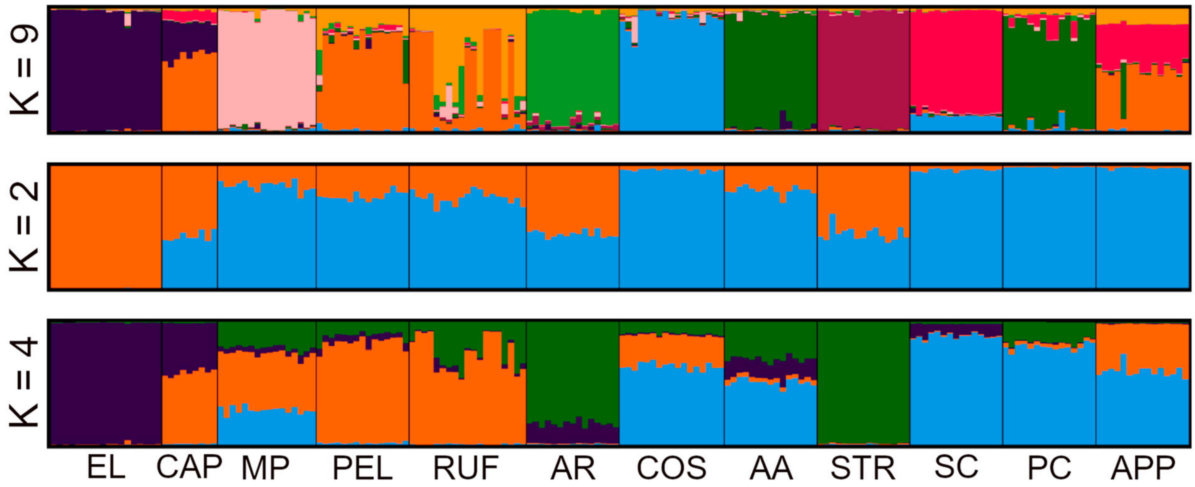

Table S1. Geographic, environmental, and sampling information of the studied populations of *Dianthus virgineus*.

Sampling site	ID	Elevation (m. a.s.l.)	Latitude (WGS84 N)	Longitude (WGS84 E)	Habitat (exposition)	Morphometry (n)	Genetics (n)	Specimens collection date (dd/mm/yyyy)	Specimens collectors	Seeds collection date (dd/mm/yyyy)	Seeds collector(s)	Voucher specimens
Isola d'Elba (Livorno)	EL	39	42.751741	10.115457	Garrigue with rosemary and rock rose on siliceous maritime cliffs (SW-W)	20	14	06/06/2020	Franzoni J. & Franzoni M.	04/07/2020	Carta A.	PI 043071, 057907, 061175-061192
Isola di Capraia (Livorno)	CAP	40	43.041384	9.844103	Garrigue on siliceous maritime cliffs (N)	2	9	10/06/2020	Franzoni J. & Peruzzi L.	29/07/2020	Franzoni J. & Giacò A.	PI 043070, 061080, 061081
Monte Pisano (Pisa)	MP	216	43.77088	10.447816	Garrigue on carbonatic rocks (SW)	20	14	03/06/2020	Astuti G. & Franzoni J.	03/07/2020	Franzoni J. & Scalzo D.	PI 061231-061250
Poggio Pelato (Livorno)	PEL	289	43.432316	10.428041	Garrigue on ophiolitic rocks (SW)	15	15	08/06/2020	Astuti G. & Giacò A.	20/07/2020	Astuti G. & Franzoni J.	PI 061120-06134
Monterufoli (Pisa)	RUF	473	43.240117	10.785517	Garrigue on ophiolitic rocks (SW-W)	20	15	19/06/2020	Astuti G. & Franzoni J.	13/08/2020	Franzoni J. & Franzoni M.	PI 061193-061212
Monte Argentario (Grosseto)	AR	500	42.409876	11.152113	Holm oak forest edge on carbonatic rocks (W)	19	15	27/06/2020	Franzoni J. & Franzoni M.	16/08/2020	Franzoni J. & Franzoni M.	PI 041621, 057908, 061213-061230
Monte Le Coste (Prato)	COS	528	43.928756	11.11943	Meadows on carbonatic rocks (E)	20	15	16/07/2020	Franzoni J. & Giacò A.	29/08/2020	Franzoni J. & Scalzo D.	PI 061155-061174
Alpi Apuane (Lucca)	AA	585	44.092893	10.208333	Meadows on marble (E)	20	15	14/07/2020	Franzoni J. & Giacò A.	03/09/2020	Astuti G. & Franzoni J.	PI 043072, 057909, 061251-061268
Stribugliano (Grosseto)	STR	970	42.858359	11.469196	Bare rocks and pine forest edge, on marl and jasper (SE)	20	15	28/07/2020	Franzoni J. & Franzoni M.	16/08/2020	Franzoni J. & Franzoni M.	PI 043074, 057910, 061269-061286
Sasso di Castro (Firenze)	SC	1060	44.127587	11.296171	Bare rocks and meadows, on basaltic rocks (SE)	20	15	16/07/2020	Franzoni J. & Giacò A.	29/08/2020	Franzoni J. & Scalzo D.	PI 061135-061154
Pania di Corfino (Lucca)	PC	1415	44.204376	10.37573	Meadows on carbonatic rocks (SW)	20	15	20/07/2020	Astuti G. & Franzoni J.	04/09/2020	Franzoni J.	PI 041622, 061101-061119
Libro Aperto (Pistoia)	APP	1874	44.158119	10.710399	High altitude meadows on sandstone (SW)	20	15	31/07/2020	Franzoni J. & Peruzzi L.	08/09/2020	Franzoni J. & Tiburtini M.	PI 043073, 061082-061100

Table S2. Pairwise F_{ST} among the studied populations of *Dianthus virgineus*. F_{ST} were calculated according to Nei (1987) on the genetic matrix (654 unlinked SNP-genotypes and 184 samples, including 3 replicates for 4 individuals). Population acronyms as in Table S1.

	**EL**	**CAP**	**MP**	**PEL**	**RUF**	**AR**	**COS**	**AA**	**STR**	**SC**	**PC**	**APP**
**EL**												
**CAP**	0.0617											
**MP**	0.0702	0.0583										
**PEL**	0.0574	0.0451	0.0331									
**RUF**	0.059	0.0559	0.0373	0.0166								
**AR**	0.0562	0.0511	0.0418	0.0299	0.0341							
**COS**	0.073	0.0697	0.0495	0.039	0.0427	0.0483						
**AA**	0.0593	0.0501	0.0408	0.0307	0.0365	0.0374	0.0444					
**STR**	0.0684	0.0648	0.0505	0.0392	0.037	0.0318	0.0542	0.0461				
**SC**	0.0698	0.0692	0.0539	0.0501	0.048	0.0537	0.0453	0.0484	0.0557			
**PC**	0.0704	0.0626	0.043	0.0392	0.0429	0.0442	0.0433	0.0288	0.051	0.038		
**APP**	0.0747	0.0613	0.051	0.0402	0.0458	0.0554	0.0425	0.0399	0.0542	0.0393	0.0273	

Table S3. Correlation among morphometric traits in the studied populations of *Dianthus virgineus*. Above the diagonal matrix, p-values of the Pearson's correlation test; in bold p-values > 0.05. Below the diagonal matrix, the coefficient of Pearson's correlation (r); in bold r > 0.8. Variable IDs are reported in Table 1.

	**1**	**2**	**3**	**4**	**5**	**6**	**7**	**8**	**9**	**10**	**11**	**12**	**13**	**14**	**15**	**16**	**17**	**18**	**19**	**20**	**21**	**22**	**23**	**24**
**1**		**0.001**	0.678	**0.002**	0.223	0.438	0.881	**0.029**	0.311	0.333	0.154	**0.047**	0.826	0.956	**0.003**	0.836	**0.044**	0.928	0.730	0.822	0.677	0.696	0.642	0.467
**2**	**0.835**		0.538	0.217	0.966	0.779	0.209	0.174	0.772	0.878	0.958	0.148	0.547	0.248	0.057	0.662	**0.010**	0.436	0.557	0.886	0.883	0.522	0.632	0.556
**3**	0.134	-0.198		0.329	0.121	0.783	0.498	0.227	0.627	0.705	0.569	0.218	0.253	0.381	0.950	0.432	0.794	0.600	0.753	0.711	0.614	0.607	0.395	0.051
**4**	0.796	0.385	0.309		**0.050**	0.079	0.072	**0.012**	**0.025**	**0.023**	**0.003**	**0.034**	0.286	0.254	**0.005**	0.286	0.327	0.446	0.092	0.990	0.183	0.548	0.839	0.544
**5**	0.380	0.014	0.472	0.577		**0.025**	**0.012**	0.363	**0.001**	**0.034**	**0.010**	0.506	**0.032**	**0.011**	0.633	0.365	0.956	0.535	0.148	0.612	0.627	0.430	0.636	0.640
**6**	0.248	-0.091	-0.089	0.525	0.641		**0.002**	**0.013**	**0.000**	**0.021**	**0.000**	0.069	**0.038**	**0.010**	0.327	0.178	0.999	0.617	**0.007**	0.928	0.239	0.067	0.952	0.368
**7**	0.049	-0.391	0.217	0.536	0.697	0.792		0.160	0.188	**0.001**	**0.001**	0.373	**0.006**	0.387	0.614	0.301	0.601	0.189	**0.026**	0.516	0.391	0.162	0.329	0.842
**8**	0.628	0.420	-0.377	0.693	0.288	0.688	0.433		**0.044**	**0.003**	**0.008**	**0.001**	0.477	0.368	**0.007**	0.408	0.391	0.438	0.079	0.991	0.242	0.250	0.854	0.491
**9**	0.320	-0.094	0.156	0.640	**0.822**	**0.856**	**0.923**	0.589		**0.000**	**0.000**	0.165	**0.012**	**0.000**	0.309	0.551	0.998	0.441	0.058	0.297	0.538	0.271	0.284	0.890
**10**	0.306	-0.050	-0.122	0.647	0.613	**0.951**	**0.818**	0.782	**0.850**		**0.000**	**0.023**	0.073	**0.020**	0.220	0.116	0.869	0.294	**0.004**	0.964	0.150	**0.050**	0.763	0.392
**11**	0.439	0.017	0.183	0.777	0.710	**0.853**	**0.830**	0.725	**0.866**	**0.890**		0.070	**0.007**	**0.013**	0.060	0.052	0.593	0.225	**0.001**	0.690	**0.049**	**0.040**	0.830	0.784
**12**	0.581	0.444	-0.384	0.612	0.213	0.542	0.283	**0.837**	0.428	0.648	0.540		0.890	0.767	**0.005**	0.444	0.469	0.979	0.239	0.881	0.461	0.434	0.963	0.450
**13**	0.071	-0.194	0.358	0.336	0.618	0.603	0.736	0.227	0.696	0.535	0.733	0.045		**0.002**	0.689	0.127	0.576	0.363	**0.023**	0.805	0.143	0.097	0.770	0.098
**14**	0.018	-0.362	0.279	0.357	0.700	0.705	**0.911**	0.286	**0.868**	0.660	0.690	0.096	0.787		0.954	0.517	0.597	0.166	0.180	0.260	0.909	0.568	0.261	0.545
**15**	0.769	0.562	-0.020	0.755	0.154	0.310	0.162	0.731	0.321	0.382	0.557	0.747	0.129	-0.019		0.564	**0.042**	0.816	0.261	0.681	0.229	0.593	0.421	0.624
**16**	0.067	-0.141	0.251	0.336	0.287	0.416	0.326	0.264	0.192	0.478	0.571	0.244	0.466	0.208	0.185		0.847	0.091	**0.002**	**0.001**	**0.004**	**0.022**	**0.032**	0.932
**17**	0.590	0.711	-0.085	0.310	-0.018	0.000	-0.168	0.273	0.001	-0.053	0.172	0.231	0.180	-0.170	0.593	-0.063		0.222	0.919	0.962	0.784	0.793	0.327	0.182
**18**	-0.029	-0.249	0.169	0.243	0.199	0.161	0.407	0.248	0.246	0.331	0.378	-0.009	0.289	0.427	-0.075	0.509	-0.381		0.269	0.534	0.419	0.616	0.631	0.729
**19**	0.111	-0.189	0.102	0.508	0.444	0.726	0.636	0.526	0.560	0.767	**0.838**	0.368	0.648	0.415	0.352	0.799	0.033	0.347		0.063	**0.000**	**0.000**	0.241	0.864
**20**	-0.073	-0.046	0.120	0.004	-0.164	-0.029	-0.208	0.004	-0.328	0.015	0.129	0.049	0.080	-0.353	0.133	**0.807**	0.015	0.200	0.552		**0.004**	0.055	**0.001**	0.935
**21**	0.135	-0.048	0.163	0.412	0.156	0.368	0.273	0.366	0.198	0.442	0.578	0.236	0.449	0.037	0.376	0.758	0.089	0.258	**0.872**	0.760		**0.002**	**0.049**	0.759
**22**	-0.126	-0.206	-0.165	0.193	0.252	0.545	0.431	0.360	0.346	0.577	0.599	0.250	0.502	0.184	0.172	0.652	0.085	0.162	**0.884**	0.566	0.785		0.366	0.608
**23**	0.150	0.154	0.271	0.066	-0.153	-0.019	-0.309	-0.060	-0.337	-0.098	0.070	0.015	0.094	-0.353	0.256	0.620	0.310	-0.155	0.367	**0.825**	0.579	0.287		0.744
**24**	0.233	0.189	0.573	0.195	0.151	-0.286	0.065	-0.221	0.045	-0.272	0.089	-0.242	0.499	0.194	0.158	0.028	0.413	0.112	-0.056	-0.026	0.099	-0.165	0.106	

Table S4. Significantly different morphometric variables among the studied populations of *Dianthus virgineus*. Significant threshold was set at 0.01. Above the diagonal matrix, the number of significantly different variables between each population pair is reported, the darker the color the higher the number of variables. Below the diagonal matrix the number IDs (Table 1) representing the variables are reported. Bold variable ID indicates significant differences resulting from Bonferroni corrected Mann-Whitney U test (Levene test p < 0.05). Non bold variable ID indicates significant differences resulting from Tukey post hoc test (Levene test p > 0.05). ns = not significant differences. Population acronyms as in Table S1.

	EL	CAP	MP	PEL	RUF	AR	COS	AA	STR	SC	PC	APP
**EL**		1	3	3	4	5	6	9	5	4	4	5
**CAP**	10		1	2	2	2	2	7	2	4	3	3
**MP**	1, **2**, 4	6		2	3	5	2	8	2	3	8	11
**PEL**	1, 4, 15	6, 10	**5**, 15		0	1	1	5	1	2	8	9
**RUF**	1, **2**, 4, 15	6, 10	**5**, 15, 19	ns		4	1	6	6	5	8	11
**AR**	1, **2**, 4, **5**, 24	6, 10	1, 10, 19, 22, 24	**5**	**5**, **7**, **9**, 12, 13		3	11	4	4	9	11
**COS**	1, **2**, 4, **12**, 15, 23	6, 10	15, 19	19	**5**	**12**, 13, 23		5	7	4	8	8
**AA**	1, **2**, 6, 16, 19, 20, **21**, 22, 23	6, 10, 13, 16, 19, 20, 22	10, **11**, 16, 19, 20, **21**, 22, 23	16, 19, 20, **21**, 23	**12**, 16, 19, 20, **21**, 23	1, 4, **5**, **7**, **11**, 13, 16, 19, 20, **21**, 23	**12**, 15, 16, 19, 20, **21**, 23		11	9	8	11
**STR**	1, **2**, 4, 15, 17	6, 10	15, 17	17	**5**, **9**, 13, 19, **21**, 22	1, 17, 19, 22	13, 17, 19, 22, 23	**7**, **11**, 13, 15, 16, 17, 19, 20, **21**, 22, 23		6	14	12
**SC**	4, 6, 13, 23	6, 10, 12, 13	**2**, **8**, 13	1, 15	1, **2**, **12**, 15, 17	1, **2**, 17, 24	1, **2**, **8**, 15	1, **2**, 16, 17, 19, 20, **21**, 22, 23	**2**, 15, 17, 19, **21**, 24		3	6
**PC**	6, 10, 19, 23	6, 10, 19	1, **5**, 6, **9**, 10, **11**, 19, **21**	1, 4, 6, 10, 15, 19, **21**, 23	1, **2** , 4, 6, 10, **12**, 15, 23	1, 4, **5**, 6, **7**, **9**, **11**, 15, 23	1, 4, 6, **8**, **9**, 10, **12**, 15	1, 4, 6, **9**, 16, 19, 20, **21**	1, 4, 6, **7**, **9**, 10, 15, 17, 18, 19, **21**, 22, 23	6, 10, 24		2
**APP**	6, 10, 16, 20, 23	6, 10, 19	1, **2**, **5**, 6, **8**, 10, **11**, 16, 19, 20, 22	1, **2**, 4, **8**, 10, 15, 16, 19, 20	1, **2**, 4, 6, **7**, **8**, 10, **11**, **12**, 15, 17	1, **2**, 4, **5**, **8**, **9**, **11**, 15, 16, 17, 23	1, **2**, 4, **8**, 10, **11**, **12**, 15	1, **2**, 4, **7**, **8**, 16, 17, 19, 20, **21**, 23	1, **2**, 6, **8**, 10, **11**, 15, 17, 19, 20, **21**, 22	1, **2**, **8**, 10, 13, 24	7, **21**	

Table S5. Spearman's correlation test results between morphometric variables and elevation in the studied populations of *Dianthus virgineus*. Results are ordered from the highest to the lowest absolute r values.

	ID	Spearman's r_S	p-value
Basal leaf width (mm)	6	-0.8601399	5.97E-04
Calyx length (mm)	16	-0.8391608	0.001192
Lower stem leaf width (mm)	10	-0.7762238	0.00466
Upper stem leaf width (mm)	8	-0.6503497	0.02591
Number of epicalyx scales	12	-0.6070213	0.03634
Corolla diameter (mm)	19	-0.59441	0.04575
Number of flowers per stem	11	-0.5649158	0.05564
Ovary length (mm)	23	-0.5454545	0.07068
Petal length (mm)	20	-0.4965035	0.1041
Petal limb width (mm)	22	-0.4475524	0.1472
Lower stem leaf length (mm)	9	-0.3706294	0.2367
Upper epicalyx scales length (mm)	13	-0.3636364	0.2463
Petal limb length (mm)	21	-0.3636364	0.2463
Upper internode length (mm)	4	-0.3286713	0.2969
Basal leaf length (mm)	5	-0.2867133	0.5273
Upper epicalyx scales mucro length (mm)	14	-0.2867133	0.3664
Plant height (cm)	1	-0.2237762	0.4849
Upper epicalyx scales width (mm)	15	-0.2167832	0.4991
Calyx teeth length (mm)	18	-0.1888112	0.5578
Number of internodes	2	-0.1225921	0.7043
Calyx width (mm)	17	-0.1118881	0.7328
Upper stem leaf length (mm)	7	-0.04195804	0.9037
Lower internode length (mm)	3	0.1258741	0.6967
Anther length (mm)	24	0.4285714	0.2992

Table S6. Morphometric distance among the studied populations of *Dianthus virgineus*. Pairwise distances were calculated as Euclidean distances of a standardized dataset without highly correlated variables (r > 0.8) and variable that did not show significant differences among populations. Population acronyms as in Table S1.

	EL	CAP	MP	PEL	RUF	AR	COS	AA	STR	SC	PC	APP
**EL**												
**CAP**	6.712											
**MP**	2.892	4.9156										
**PEL**	4.4524	5.6664	3.0183									
**RUF**	4.4136	6.5143	3.5145	2.5913								
**AR**	5.7018	5.3357	4.0668	3.6835	4.1548							
**COS**	4.9742	5.8458	3.4818	3.1801	2.4609	4.521						
**AA**	6.0985	8.8457	5.6814	6.3346	5.1969	6.4487	4.984					
**STR**	4.8723	5.9136	4.194	3.6272	3.2764	3.3829	4.4443	6.2776				
**SC**	3.3063	6.9444	2.6213	3.9677	4.1811	5.581	3.9117	5.1833	5.6974			
**PC**	4.8613	7.9757	4.4383	5.1304	5.6952	5.984	5.6396	5.4961	6.4124	4.3198		
**APP**	3.9363	7.8481	4.1867	5.2307	5.5202	5.8544	5.4685	5.0583	5.9544	3.8275	2.7721	

Table S7. Elevation distance among sampling sites of the studied populations of *Dianthus virgineus*. Pairwise distances were calculated in meters. Population acronyms as in Table S1.

	EL	CAP	MP	PEL	RUF	AR	COS	AA	STR	SC	PC	APP
**EL**												
**CAP**	1											
**MP**	177	176										
**PEL**	250	249	73									
**RUF**	434	433	257	184								
**AR**	461	460	284	211	27							
**COS**	489	488	312	239	55	28						
**AA**	546	545	369	296	112	85	57					
**STR**	931	930	754	681	497	470	442	385				
**SC**	1021	1020	844	771	587	560	532	475	90			
**PC**	1376	1375	1199	1126	942	915	887	830	445	355		
**APP**	1835	1834	1658	1585	1401	1374	1346	1289	904	814	459	

Table S8. Geographic distance among sampling sites of the studied populations of *Dianthus virgineus*. Pairwise distances were calculated in kilometers. Population acronyms as in Table S1.

	EL	CAP	MP	PEL	RUF	AR	COS	AA	STR	SC	PC	APP
**EL**												
**CAP**	43.70											
**MP**	117.54	104.16										
**PEL**	82.18	77.51	37.13									
**RUF**	91.39	106.38	69.05	44.79								
**AR**	120.61	160.35	167.99	137.54	99.39							
**COS**	170.06	171.28	76.27	93.79	83.85	165.96						
**AA**	147.33	122.11	44.11	76.33	113.10	211.66	102.37					
**STR**	150.19	180.87	150.71	131.15	86.36	60.23	123.10	194.10				
**SC**	199.27	199.82	101.62	122.47	112.23	188.26	29.22	120.39	139.84			
**PC**	161.73	140.38	48.13	84.74	114.85	214.23	87.61	22.18	190.47	102.16		
**APP**	167.47	155.39	51.38	85.35	100.75	197.25	51.73	55.99	164.95	64.88	37.36	
